# Supplementary material for: Evaluation of the Polysaccharide “Immeran” Activity in Syrian hamsters’ Model of SARS-CoV-2
Source: Viruses. 2024 Mar 9;16(3):423. doi: 10.3390/v16030423 (PMC10976179; doi:10.3390/v16030423)
Supplement: Supplementary file 1 [file viruses-16-00423-s001.zip › viruses-2880183-supplementary.pdf]

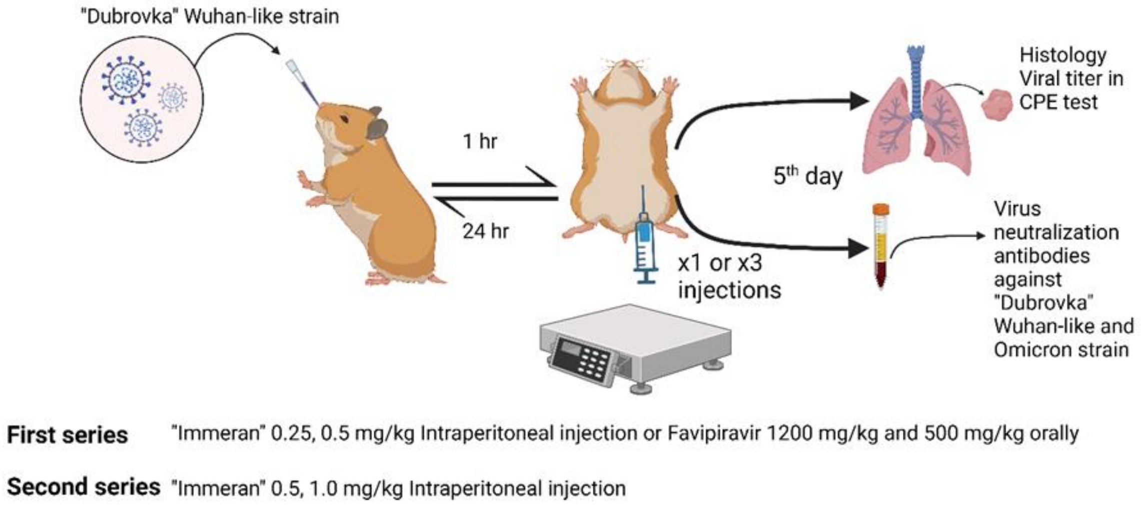

Figure 1. Flowchart of animal experiments 1 and 2.

Table. Cumulative severity scoring from H&E stained slides of lungs from golden hamsters with SARS-CoV-2-associated interstitial pneumonia and different treatment dosage and regimes using “Immeran” drug.

Criterion for assessing the severity of the inflammatory process in the lungs	Animal group																													
	1 viral control group					2 one intraperitoneal injection 0.5 mL of 250 μg/kg of “Immeran” 24 hours before infection					3 one intraperitoneal injection 0.5 mL of 500 μg/kg of “Immeran” 24 hours before infection					4 one intraperitoneal injection 0.5 mL of 250 μg/kg of “Immeran” 1 hour after infection and four days once every other day					5 one intraperitoneal injection 0.5 mL of 500 μg/kg of “Immeran” 1 hour after infection and four days once every other day					6 Favipiravir orally 1200 mg/kg 1 hour before infection, then 500 mg/kg twice a day for 4 days				
Animal № in group	1	2	3	4	5	1	2	3	4	5	1	2	3	4	5	1	2	3	4	5	1	2	3	4	5	1	2	3	4	5
Number of animals in group without inflammatory changes in lungs	0					0					1					2					3					0				
Prevalence of inflammation (in points from 0 to 3)	3	3	2	2	3	3	2	3	2	3	2	0	1	2	3	0	1	1	0	2	1	1	0	0	0	1	2	2	1	1
Extensive airless foci of pneumonia (in points from 0 to 3)	3	3	2	2	2	3	2	2	3	3	1	0	1	1	2	0	1	0	0	1	0	1	0	0	0	0	1	1	0	0
Small scattered foci of pneumonia (in points from 0 to 3)	2	2	3	3	3	1	3	2	2	3	2	0	1	1	2	0	2	2	0	2	1	1	0	0	0	1	2	2	1	1
Bronchitis, bronchiolitis (in points from 0 to 3)	2	2	2	2	2	2	2	2	3	2	2	0	1	2	2	0	1	1	0	1	1	1	0	0	0	1	1	1	1	1

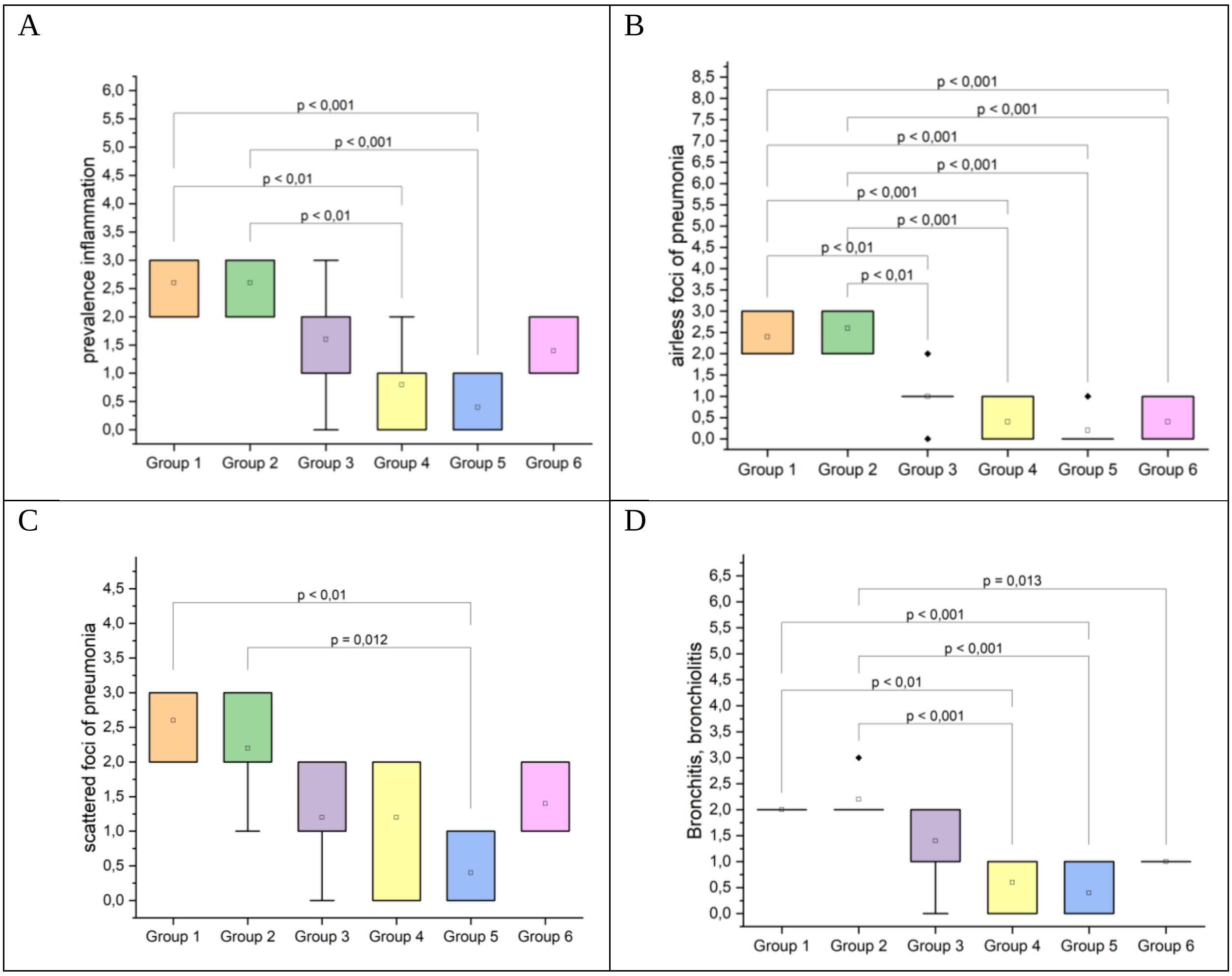

Figure 2. Statistical analysis of data from Table 1. Statistical comparisons between groups were done using unpaired Student's t-test ($p<0.05$, n=30). Group 1 - viral control group, Goup 2 - one intraperitoneal injection 0.5 mL of 250 µg/kg of "Immeran" 24 hours before infection, Group 3 - one intraperitoneal injection 0.5 mL of 500 µg/kg of "Immeran" 24 hours before infection, Group 4 - one intraperitoneal injection 0.5 mL of 250 µg/kg of "Immeran" 1 hour after infection and four days once every other day, Group 5 - one intraperitoneal injection 0.5 mL of 500 µg/kg of "Immeran" 1 hour after infection and four days once every other day, Group 6 - Favipiravir orally 1200 mg/kg 1 hour before infection, then 500 mg/kg twice a day for 4 days.

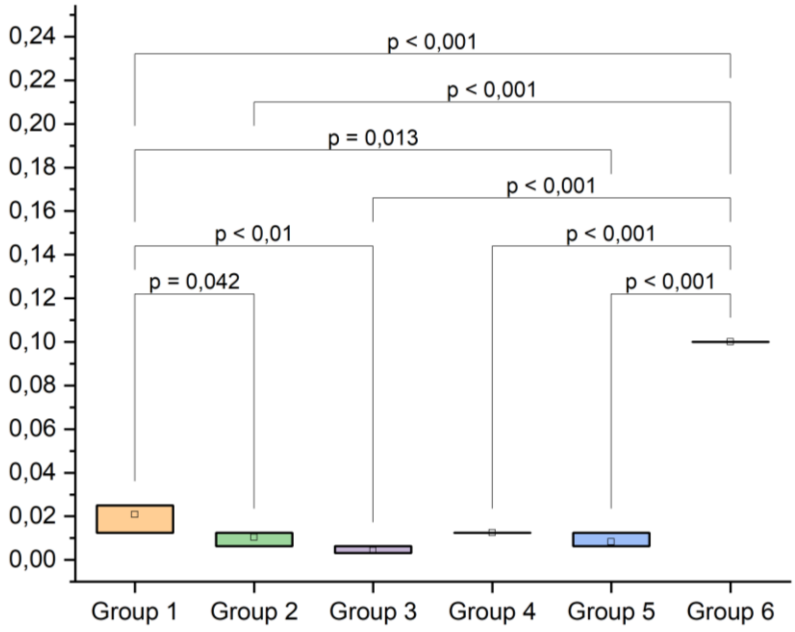

Figure 3. Statistical analysis of virus titerStatistical comparisons between groups were done using unpaired Student's t-test ($p<0.05$, $n=30$). Group 1 - viral control group, Goup 2 - one intraperitoneal injection 0.5 mL of 250 µg/kg of "Immeran" 24 hours before infection, Group 3 - one intraperitoneal injection 0.5 mL of 500 µg/kg of "Immeran" 24 hours before infection, Group 4 - one intraperitoneal injection 0.5 mL of 250 µg/kg of "Immeran" 1 hour after infection and four days once every other day, Group 5 - one intraperitoneal injection 0.5 mL of 500 µg/kg of "Immeran" 1 hour after infection and four days once every other day, Group 6 - Favipiravir orally 1200 mg/kg 1 hour before infection, then 500 mg/kg twice a day for 4 days.
